# Mental health nurses’ support to caregivers of older adults with severe mental illness: a qualitative study

**DOI:** 10.1186/s12912-015-0087-5

**Published:** 2015-06-24

**Authors:** Marian I. Zegwaard, Marja J. Aartsen, Mieke H. F. Grypdonck, Pim Cuijpers

**Affiliations:** Altrecht Mental Health Care, Gedachtengang 1, 3705 WH Zeist, The Netherlands; Faculty of Social Sciences, VU-University Amsterdam, de Boelelaan 1081, 1081 HV Amsterdam, The Netherlands; Faculty of Nursing Science, Ghent University, St. Pietersnieuwstraat 33, B-9000 Ghent, Belgium; Faculty of Clinical Psychology, VU University Amsterdam, Van der Boechorststraat 1, 1081 BT Amsterdam, The Netherlands

**Keywords:** Older adults, Severe mental illness, Mental health nurses, Nursing practice, Caregiver support

## Abstract

**Background:**

Literature has shown the serious impact of severe mental illness on the daily life of caregivers. We studied reported caregiver support practices by mental health nurses for use in the development of a nursing intervention. We aimed to explore current caregiver support practices by mental health nurses.

**Methods:**

Twenty-one participants completed semi-structured interviews, and 17 participants attended two focus groups. All interviews were audio-taped, transcribed and coded for qualitative analysis.

**Results:**

The diversity in caregiver support could best be described by three prototypes: the tolerator, the preventer and the concerner, representing three approaches of involvement with caregivers. At one end of the spectrum are mental health nurses (MHN) who are essentially only concerned with the wellbeing of the care recipient and see the caregiver as a potential impediment in reaching the client’s goals. We call these the tolerators. At the other end of the spectrum are the MHNs who see the caregiver and the care recipient as inextricably connected with each other. In these cases the MHN directs her/his intervention towards both the informal caregiver and the care recipient. We call these the concerners. In the middle position are MHNs who realize that caregivers are important agents in the achievement of the client’s goals, and therefore consider preventing them from becoming overburdened as an important goal. We call these the preventers.

**Conclusions:**

Based on the extent to which the MHNs believe that the informal caregiver plays a necessary role in the client’s support system, and the degree to which they feel responsible for the caregiver’s wellbeing, three MHN prototypes can be distinguished. These prototypes determine how the nurses’ vision directs their understanding of their role and responsibilities and the content of their behaviour. This implies that a change in behaviour needs to be preceded by a change in vision. Therefore, promoting family support cannot be achieved by one-size-fits-all-programmes.

## Background

Severe mental illness, including bipolar disorder, schizophrenia and depression, is associated with a substantial loss of quality of life [1], increased rates of mortality and morbidity, high levels of service use and huge economic costs [2]. However, it is not only the patients themselves who suffer. With the deinstitutionalization of care which has taken place in recent decades, a growing number of patients are cared for at home by relatives. These caregivers spend a significant amount of time and energy in caring for their loved ones, despite the large emotional burden and the impact on their personal goals [3–12]. Given these consequences, it is not surprising that caregivers have a significantly increased risk of becoming overloaded [7, 12–14], which can severely impair their quality of life, potentially leading to withdrawal from caregiving.

Mental health nurses constitute one of the core professional groups involved in the treatment and support of older psychiatric patients and their caregivers. They meet the caregivers of patients with severe mental illness in their everyday work. Nursing interventions to address the essential needs of caregivers can however, be considered to be still in their infancy [15, 16]. These include information about the illness and treatment; training in skills to cope with the illness and its implications for the family; and support for themselves. For the development of a nursing intervention that matches the complex nature of both patient and caregiver needs, it is important to identify and refer to existing nursing practices [17, 18].

We decided, therefore, to conduct a qualitative study with the aim of gaining insight into the current practice of mental health care nurses’ support to caregivers of older adults with severe mental illness. We studied reported caregiver support practices in two large mental health care organizations in the Netherlands. This study was embedded in a larger research project focusing on the development and evaluation of a theory–based nursing intervention to support caregivers of older adults with severe mental illness.

## Method

A pragmatic approach based on the constant comparative method together with purposive sampling was the core of this qualitative study and analysis [19–22].

### Participants

Mental health nurses from two large mental health care organizations in the Netherlands were invited to participate in the study. The MHNs provided care to older community dwelling or hospitalized clients with a severe mental illness and their families. The clients were at least 60 years of age.

To ensure that the MHNs’ perception and content of caregiver support were explored in diverse situations, maximum purposive sampling was used [20–22]. Consequently, MHNs with diverse characteristics were included (gender, education level, and years of experience) and with different contexts (hospital vs community, organization) were involved. These characteristics may influence the perspectives and content of caregiver support.

As graduate nurses (i.e., with a bachelor’s degree) are the in majority in the teams their personal views and experiences concerning caregiver support carried the greatest weight. It was considered important that the graduate nurses would feel at ease during the interviews. Therefore they were free to choose to participate either in an individual interview or a focus group. Based on the level of education of the applied and academic mental health nurses we expected a “senior” best practice on caregiver support. This and the fact that they are used to sharing views on the topic in public were leading for an allocation in a focus group.

In recruiting the participants the researcher explained the aim and design of the study to four teams, two in each hospital, and handed out a written information bulletin. One week after the team meeting, the researcher invited the MHNs to participate, and an appointment was made for either an individual interview or a focus group. More MHNs volunteered than required for the study, thus ensuring saturation. The participants were assured of anonymity and were free to leave the study whenever they wished. In total, 38 MHNs agreed to participate and they all remained in the study until the data collection was completed. Table 1 gives the characteristics of the participating MHNs.

**Table 1:** Characteristics of the study sample

Characteristics		*N* = 38
Gender	Male	14
	Female	24
Age	20–30	6
	30–40	5
	40–50	15
	50+	12
Education level	Student	4
	Graduate (Bachelor)	24
	Applied Master’s Degree	5
	Scientific Master’s Degree	5
Department	Psychiatry Ambulant	28
	Psychiatry Hospital	10
Years of experience	MHN (*n* = 34)	19(mean)
		3–40 (range)
	Students (*n* = 4)	Traineeship

**Table 2:** Summarized results

	Tolerator	Preventer	Concerner
Vision on support	● Relationship only with client	● CG is an ally of the MHN	● Focus is on fostering the quality of life: Wellbeing of CG, the client and their interrelatedness
	● Care for client is main focus		
	● CG is potential obstacle to reaching client goals		● Wellbeing of CG and Cl is interrelated
	● Assumption that family ties are irreversibly weak		● Systemic approach is needed
Interpretation of role and responsibility	● Responsible for treatment environment	● To support CG as teammate by preventing excessive burden in order to prevent drop out	● Support both CG and Cl
	● Modelling of appropriate CG behaviour	● Observe CG-client relationship and offer practical solutions	● Focus on relieving the suffering of both CG and the Cl and reaching full potential of both
	● CG Problems referred to other professionals if needed	● Avoid being dragged into the situation	● Presence comes before problem-solving
Acknowledgement of relationship with the caregiver	● Keeping distance	● Equal, professional and trusting relationship	● Aims at building a trusting, reciprocal, non-hierarchical relationship with CG and Cl
	● Contact only in order to gain access to client and gain information about client illness (manifestation)	● Maintaining the CG-MHN relationship is fostered by recognizable narratives as well as the CG’s empathy for the client’s situation and by working together	● CG is viewed as expert
		● Relationship is hampered by CG refusing assistance	● Relationship is based on understanding the CG as a person
			● Present without prejudices
Defining CG needs	● Defining CG needs is not an issue	● Focus on problems with caregiving tasks and not on CG emotions	● Emotional impact of the mental illness on expectations, treat to integrity, dreams and life course of both the caregiver and the client
		● No systematic assessment of CG needs	● Assess the impact on the interrelatedness and mutual dependence of the CG and Cl
		● Support based on assumptions about CG needs rather than facts	● Assessment by open and empathic listening to the CG narratives
			● Assessment in the absence of Cl is needed
Interventions that meet the CG’s needs	● Information and modelling concerning behaviour preferred to reach clients’ goals	● Support by problem oriented and instrumental advice	● Presence is most important intervention
		● Listening to CG stories	● MHN is mediator rather than decision maker
		● Acts more pro-actively as relationship deepens	● Improvement of mutual communication, problem-solving strategies and personal development
		● In the case of CG-client conflict in goals CG support is left to colleague	

*CG* caregiver, *Cl* client

### Data collection

Data were collected through individual semi-structured interviews conducted with 21 MHNs, as well as via two focus groups with 10 and 7 MHNs respectively. Both the individual interviews and the focus groups were designed to elicit retrospective descriptions of cases in which the MHNs supported caregivers. The individual interviews aimed to gain in-depth insight into the respondents’ personal views and experiences concerning their caregiver support. The focus groups provided an opportunity to share views on the topic of caregiver support, ensuring diversity of perceptions. This type of data triangulation facilitated a deeper understanding of the MHNs’ views on how they provided support to caregivers. The data-collection took place between November 2008 and October 2009. The interviews with the individual MHNs were conducted in a private area adjacent to their office.

The individual interviews were conducted by the first author (MZ) and focus groups were facilitated by the first (MZ) and second author (MA). The individual interviews lasted 60–90 min. Both focus groups were held in a meeting room also adjacent to the MHN’s office. The first focus group comprised ten nurses with post-graduate degrees (Master’s). The second focus group comprised undergraduate students (4) and graduate nurses (3). Both focus group meetings lasted 90 min. Except for one of the individual interviews, all interviews and the focus group discussions were tape recorded. Written consent was obtained from the participants.

One week before the interview a case description was sent to all participating nurses. This case described the lived experience of a caregiver who had been interviewed in a previous study conducted by the researcher. At the start of the interview, the MHNs were encouraged to tell their story by an open-ended reflective question: “What are your first thoughts about the case you read while preparing for this interview?” The intention was to help the MHNs to more easily verbalize their experience in daily practice. After the reflection on the case, the MHNs were asked to describe their own cases and talk about the attention they give to caregivers in those cases.

A topic guide was used to gain further insight into daily practice. This topic guide was based on the literature review [3] and the study about the lived experience of caregivers preceding this study [23]. Topics were the MHNs’ opinion about what is important in the support of caregivers, caregiver-MHN collaboration, the MHNs’ opinion about the caregivers’ needs and the content of the MHN caregiver support. The latter questions focused on barriers and opportunities in supporting the caregiver. The initial question was “What would you do to assess the caregiver’s situation”? The participants were invited to describe the content of their best practices by giving details of events, situations and conversations with the caregiver. Both the individual interviews and the focus group discussions followed the natural course of a conversation. Field notes concerning impressions gained during the individual interviews and focus group discussions, as well as information given after the tape recorder was turned off were written down immediately after the interviews.

### Data analysis

The analysis of the individual interviews was conducted in a cyclical process in which two complementary and intertwined strategies were used, namely coding and theoretical thinking [22]. A research team of three members (MIZ, MA, MG), including both interviewers (MZ, MA) was involved in the entire process of data analysis. Data collection and data analysis were executed iteratively. Accordingly, in a first step, two researchers (MZ and MG) read ten transcripts in full. Analytical thoughts and ideas with respect to the data were discussed in order to reach an understanding of the respondents’ point of view [24]. Notes were made about the initial concepts pertinent to the interviews [19, 21, 25]. In a second step, 11 further interviews were conducted, and the already formulated concepts and themes were juxtaposed with new material [19]. During the meetings, two researchers (MZ, MG) constantly compared their interpretations of the data and worked towards consensus about the interpretation of possible meanings thus guiding the constant comparative analysis. In order to arrive at a more thorough understanding of the participants’ perspectives and experiences the researchers discussed commonalities, differences, and explanations for discrepancies between interviews. Comparing and contrasting elements within and between cases enabled them to find out what was shared and what was different. A reflection on this analysis was described, text parts were coded and a code tree was developed. Coding was supported by the software program MAXqda. In order to further strengthen researcher triangulation, a third researcher (MA) was involved in the analysis of the individual interviews and a fourth independent researcher (JG) was involved in the analysis of the focus groups. The third researcher critically examined the conclusions based on the interpretation of the data. During triangulation meetings all three researchers worked together in checking the interpretation of the data against existing data and new materials. New codes were added and the code-tree was restructured in accordance with theoretical insights. Concepts were further categorized and main themes were identified [19, 20]. Relations between categories and between themes were established and categories developed. It appeared that the diversity in nursing practices could best be described by making use of three prototypes. These prototypes represent three degrees of involvement with caregivers.

To further strengthen the data analysis [26], the data from the two focus groups was reviewed by a fourth researcher. The fourth researcher (JG) read the verbatim interviews and was then asked to read the results section. Both the first and this fourth researcher separately categorized all text statements that were attributable to one of the three types. They compared their interpretations of the fragments they agreed on and discussed their differences regarding allocation to prototype. Differences in opinion about the allocation could be explained by the fact that the MHNs exhibited features of the two adjoining prototypes on the spectrum. This final check showed high inter-rater agreement.

### Ethical considerations

Ethical approval was obtained from the Institutional Research Ethics boards of Altrecht Mental Health Care and Symfora Mental Health Care. In the study, written informed consent was obtained from each participant at the beginning of the initial interview after they were given information about the study and informed that they could withdraw at any time. With permission, interviews were recorded using a digital recorder and later transcribed in full. Confidentiality regarding the collected data was assured.

## Results

The MHNs acknowledge the importance of family relationships, “*nobody can do without a family”,* but nonetheless, their caregiver support varies considerably. Based on the extent to which the MHNs believe that the informal caregiver plays a necessary role in the client’s support system, and that caregiver wellbeing is important, three MHN prototypes can be distinguished. At one end of the spectrum, we have the MHN who sees the caregiver and the care recipient as inextricably connected with each other. In these cases the MHN directs her/his intervention towards both the informal caregiver and the care recipient. We call these the concerners. At the other end of the spectrum we see an MHN who is basically only concerned with the wellbeing of the care recipient and considers the caregiver to be a potential obstacle in reaching the client’s goals. We call these the tolerators. In the middle position are MHNs who realize that caregivers are important agents in attaining the client’s goals, and consider preventing them from becoming overburdened as the main goal of their support. We call these the preventers.

Notwithstanding the distinction of three prototypes, in practice pure prototypes are rarely seen. Instead, the MHNs may also exhibit features of an adjoining prototype, depending on the specific situation. For reasons of clarity we describe only the three pure prototypes. Each prototype description begins with a presentation of the MHNs’ essential understanding of caregiver support. Based on this essential understanding, we describe their interpretation of their role and responsibilities and the manner in which the MHNs perceive the MHN-caregiver relationship Table 2. Finally, the MHNs’ assessment of caregiver needs and the nature of the interventions that fit their respective role conception are described.

### The three prototypes

#### *The tolerator* (for illustrative quotes see Table 3)

**Table 3:** The tolerator

Aspects	Illustrative quotes^a^
View of support	*I think I have considerable responsibility and I know what I’m doing, because it is about the wellbeing of the client*
Int 39	
Interpretation of role and responsibilities	*It’s a minefield. I think it has something to do with the culture. It is the client that is important and you cannot just involve the entire support system. And where does the support stop? It’s the client that matters and to what extent do you give family support? Do you talk about the client while talking with the caregiver? Do you need to ask for permission every time? I mean there might be tension because people feel patronized. Or the family member is pressured because of their history with the client and family members are uncertain about what will happen next. As a professional you are already happy when you have enough time to do your job properly for the client, and support of caregivers would be felt as an additional burden*
Int 59	
MHN-caregiver relationship	*In the beginning I pretend that I’m interested in the caregiver also and I am a little interested because I have to gain the confidence of not only the client but also the caregiver. I must have permission to be alone with my client.*
Int 39	
Defining caregiver needs	*I believe I’ve done it only once I think; talking with the husband but that is an exception. No, I really focus on the needs of the client*
Int 51	In the case of relapse prevention; *“Over time I just had to learn that caregivers do not want to take over your role, yet they do see the early signals and you can still take that very seriously”*
Int 39	
Interventions	*“As an MHN I have to set a good example of how to deal with individual clients. I am used to doing this because I set examples in groups of clients on the ward where I worked as a nurse”*
Int 39	

^a^Illustrative quotes have been slightly edited to improve readability

The tolerator focuses almost exclusively on reaching the treatment goals set for the client. A tolerator does not pay serious attention to the caregiver because the caregiver is seen as a potential obstacle to providing care for the client. Tolerators also assume that because of the severity and long-lasting character of the mental illness, the caregiver may lose interest in the situation and may no longer wish to be involved. They consider this loss of contact between client and caregiver and friends an irreversible fact. In the eyes of the tolerator, the caregiver lacks the skills and/or understanding to cope with the client’s condition and behaviour. The tolerator also believes that standard provision of caregiver support is experienced as unwelcome by clients.

As a consequence, the tolerator builds a relationship with the client only. The attention given to the caregiver is meant only to obtain the caregiver’s “trust and confidence” in order to gain and maintain free access to the client and to the necessary information.

#### Interpretation of role and responsibilities

As the tolerators hold themselves responsible only for the treatment of the client’s mental illness they feel responsible for organizing the appropriate treatment environment. Tolerators assume they are improving the treatment environment by demonstrating professional interaction with the client that the caregiver can imitate. The MHN believes that the caregiver can benefit from this modelling. The tolerators mention several reasons why they solely focus on client goals. First they refer to a work culture in which caregiver support is not considered an important professional activity and second, in their opinion, caregiver support adds to the already existing demanding work situation. The time spent on the caregiver is not considered justified, and if a need for help is detected, the caregiver is referred to other professionals such as a psychologist.

#### Acknowledgement of the relationship with the caregiver

As the interaction between client and caregiver is considered a possible threat to achieving the client’s goals, the tolerator avoids engaging with the caregiver. The tolerator’s contacts with the caregivers are meant to gain the trust of the caregiver in order to obtain access to the client. Besides gaining trust, the tolerator also wants to receive additional information from the caregiver about the client’s illness and treatment. Even in problematic client-caregiver interaction, the tolerator always chooses the side of the client. They never intervene in the relationship between the client and caregiver.

#### Defining caregiver needs

The tolerator does not focus on the needs of the caregiver. Although the main focus is on the client’s needs the tolerator does listen to the caregiver’s story but then hears and interprets this story from the perspective of the diagnosis and the treatment of the client’s problems.

#### Interventions that meet the caregiver’s needs

The intervention of the tolerator focuses on providing information to the caregiver about modelling the appropriate attitude and behaviour needed to help the client. Caregivers are also allocated a function in the early detection of relapse, although the tolerator first had “to learn” to see the role the caregivers could play in this.

#### The preventer (for illustrative quotes see Table 4)

**Table 4:** The preventer

Aspects	Illustrative quotes^a^
View of support	*It is very important to involve the family. Because you know it is not possible without the partner and the system. For the family it is very important that they are heard and seen and work together*
Int 42	
Interpretation of role and responsibilities	*And the caregiver, she was really burdened as far as I could see. There was considerable interaction between them. They told me that the general practitioner asked them why they didn’t get a divorce. Well, that also crossed my mind but I am not in a position to say such things. But they choose to be together and that made things awful. I asked my superior for advice, because I had never had any training on this topic nor did I have any experience in conversations about caregiver-client relationships. I tried to offer them some space by organising respite care but I couldn’t get any further. My superior formulated it as the caregiver’s choice. She said; ‘you gave them advice and if they do not learn from it, it is their choice and there is nothing you can do about it’. So in the end I gave up, I could not help them but I felt bad*
Int 44	
MHN-caregiver relationship	*I assume that when there is confidence and mutual trust, then they will more frequently ask questions for their own reassurance. This also influences the client*
Int 49	
Defining caregiver needs	*Everyone has a family. So to some extent you have to know things about family ties. You need to know what activity you can expect from family and what information you can give them*
Int 48	
Interventions	*They do not communicate but argue. I arrange the medication so they do not have to argue about this*
Int 43	*I explain things about the illness so they understand*

^a^Illustrative quotes have been slightly edited to improve readability

Occupying the middle position on the spectrum are the MHNs whose approach is based on the belief that the client needs the caregiver. The MHN focuses on the goals set for the client and is at the same time aware that the caregiver is an important agent in achieving these goals. The preventer acknowledges that the caregiver’s “hands-on” expertise is an important source of information and very useful in the process of diagnosis and treatment of the client’s problems. Therefore, the preventer is careful about anything that might place an excessive burden on the caregiver.

#### Interpretation of role and responsibilities

The preventers interpret their role as being the team leader and consider the caregiver a teammate in caring for the client. This teammate must be prevented from dropping out, and therefore the preventer focuses on solving caregiver problems in order to prevent excessive caregiver burden.

One of the sources of burden is the observable friction or misunderstanding within the caregiver-client relationship. Preventers therefore closely observe the client - caregiver relationship, and although they take account of the emotional needs of both caregiver and client, they mostly offer practical solutions like respite care instead of intervening in the interaction. Due to role ambiguity and uncertainty about their own skills, the preventers are afraid of “being dragged into the situation”. Preventers refer to situations of this nature in terms of “problematic family” and feel that because they are not a therapist, they cannot be expected to provide a solution. In these situations the preventer often responds with personal judgements.

#### Acknowledgement of the relationship with the caregiver

Initially, the preventers invest in building a relationship with the client. Fearing a conflict of loyalty, they preserve a professional distance in their contact with the caregiver. Encountering the caregiver is seen as something that comes naturally. Preventers invest in building an equal and trusting relationship with the caregiver. Recognizable narratives as well as the caregiver’s empathy with the client’s situation are important conditions for building this relationship. By working together the relationship is deepened, and with increasing mutual trust, the caregiver is more willing to continue to support the client. The appreciation of the caregiver for the support as expressed in “*I don’t have to face the responsibility alone”* is seen as confirmation that the preventer is giving the right kind of support. If the caregiver refuses assistance, this may be interpreted as a rejection of the preventer as a person. In such cases the relationship tends not to develop successfully.

#### Defining caregiver needs

In assessing the caregiver’s needs, the preventer concentrates mainly on problems with caregiving tasks. Due to client loyalty, the assessment of caregiver needs almost always takes place in the presence of the client. The preventer does not systematically assess the needs of the caregiver; they mostly operate from assumptions rather than from facts. The preventer is friendly, empathic and open to questions and narratives from the caregiver, but hardly ever focuses on the emotions of caregiving. Focusing on caregiver burden would complicate the interpretation of signals of emotional distress and delay adequate coaching. Rather than exploring emotional needs, the preventer prefers to adopt a wait-and-see approach. The quote about defining the caregiver needs in Table 4 illustrates how uncertainty on the part of the preventer in how to respond to caregiver needs can be interpreted as “respecting the autonomy of the client”.

#### Interventions that meet the caregiver’s needs

In order to reduce the caregiver’s burden, the preventer supports the caregiver with problem-oriented and instrumental advice or solutions such as respite care and homecare. Time after time the preventer listens to the caregiver’s stories. When the preventer and the caregiver have become more familiar with each other, the MHN offers support more pro-actively. When the preventer experiences a conflict between the client’s goals and caregiver needs, the preventer often chooses to delegate caregiver support to a colleague.

#### The concerner (for illustrative quotes see Table 5)

**Table 5:** The concerner

Aspects	Illustrative quotes^a^
View of support	*To boost the caregiver’s strength, to help the client become calmer, less angry, less aggressive. Yes it sounds silly, but to increase the quality of life of both a little. We cannot heal the past but we can help to make things better for today and for a positive future together”*
Int 51	
Interpretation of role and responsibilities	*I think you physically must be there for these people, you must get to know them. You must know the system and how it works and you should be there for them. These people with psychiatric illnesses, they have gone through a lot. That is why the emphasis is on being there, making contact*
Int 52	
MHN-caregiver relationship	*By doing your best to understand why someone does what he does, you learn to know the person behind the caregiver en you get closer to that person*
Int 58	
Defining caregiver needs	*I want to know everything. How they manage their situation, if there are any children. What kind of support they give*
Int 45	
Interventions	*When there are things the caregiver does not feel capable of doing, I might take over some concrete tasks for the time being. I have to pay close attention and listen carefully because caregivers differ in pulling the strings. Some want to arrange everything themselves while others need help. It is awfully important to allow them this choice and that is what I do*
Int 45	

^a^Illustrative quotes have been slightly edited to improve readability

The concerner focuses on the well-being of the caregiver, the client and their interrelatedness. In the concerner’s view, the illness and its consequences are not something the caregiver or the client asked for and they have already invested a lot in staying together. Therefore, concerners feel they should foster the quality of their life together. The concerner does not construct the individual well-being of the caregiver and that of the client as opposites. The way both live their life, their mutual solidarity and the quality of their relationship are all affected by the illness and its symptoms. They believe communication between client and caregiver is important and that a systemic approach is needed in which particular attention and support is given to quality of life issues concerning the caregiver, the client and the solidarity between these two persons.

#### Interpretation of role and responsibilities

Because of their acknowledgement of the interrelatedness of caregiver and client, the concerners never hesitate to support both. The concerner feels a responsibility to relieve the suffering of both caregiver and client and to help both individuals reach their full potential. In the concerner’s opinion, there is no urgency to solve all problems, but a wish to offer empathic presence, attention and closeness. Concerners do not question the legitimacy of their support but they are uncertain about what so-called professional support must include to be effective. Therefore the concerners share their experiences with their colleagues. In doing so they enhance their skills and expertise and they take their share of professional responsibility in collaboration with colleagues. The latter help the concerners to manage their own expectations about the effectiveness of their support and this prevents them from being dragged into the situation.

#### Acknowledgement of the relationship with the caregiver

The concerner aims at building a trusting relationship with both the client and caregiver rather than being problem or task-oriented. These trusting relationships are reciprocal, non-hierarchical relationships, in which caregivers are viewed as experts and their experience, strengths and resources are valued: “(int56) *who knows more about yourself than yourself?*” During relationship building the concerner wants to meet the person behind the caregiver and find out what causes the (relational) suffering. By giving room to the caregiver’s narrative, the concerner learns about the meaning of changes in the caregiver’s life and about how the mutual caregiver-client dependency makes them more or less condemned to each other. The concerner is present without prejudices. In order to avoid a possible conflict of loyalties the concerner discusses mutual expectations, and although feelings of sympathy for the caregiver should not determine the relationship, building a confidential relationship is easier when no animosity is felt. The concerner seeks creative solutions to the application of rules and legislation regarding autonomy and privacy. Concerners do not need the caregiver’s gratitude as an incentive.

#### Defining caregiver needs

The concerner observes the emotional impact of the mental illness on expectations, dreams and life course of both the caregiver and the client. The concerner assesses caregiver needs, recognizing that caregivers also have to resolve their own emotional burden, preserve the integrity of their own lives, and fulfil their personal hopes and dreams. Concerners also assess the impact of the illness on the interrelatedness and mutual dependence of the caregiver and client. The assessment of the caregiver’s needs is a process of trying to get to know and understand the lived world of caregivers and how they cope with the serious implications for their own lives and their changed interaction with the client. With an open mind the concerners listen to the caregiver’s narrative, try to empathize with the caregiver’s thoughts, fears, joy and suffering. They also want to know who else is involved in caregiving. In order to learn more about the caregiver they sometimes invite the caregiver for a conversation in the absence of the client.

#### Interventions that meet the caregiver’s needs

The most important “intervention” is to be present and listen attentively to the caregiver’s narratives. For instance when a husband tells the MHN he has been very aggressive towards his wife, he/she “*wants to know where this frustration came from”*. The concerners support the caregiver through continuous consultation. The concerners consider themselves as mediators and not decision makers. The caregiver is in charge and when the need arises, the caregiver temporarily lets the concerner take over some tasks. Besides problem-solving strategies, the concerners also try to improve communication between caregiver and client, for instance by mirroring. The concerner encourages personal development so that the caregivers can handle difficult situations and make decisions about their lives and their shared life with the client. Despite their understanding of the situation the concerners remain uncertain about the effects of their intervention on the wellbeing of the caregiver.

## Discussion and conclusion

### The findings

The aim of the study was to explore and interpret current practices in mental health care nurses’ support to caregivers. These show great diversity. We can identify three prototypes in the data: the tolerator, the preventer and the concerner. These prototypes represent three approaches to being involved with caregivers. For each type we described the MHN’s vision, their interpretation of their role and responsibilities, their acknowledgement of the relationship with the caregiver, how the caregiver’s needs are defined and the interventions used in relation to the caregiver.

The tolerator considers the caregiver a possible obstacle to organising the appropriate treatment environment for the client. Attention is paid to the caregiver in order to gain access to the client, to obtain information about the client and to influence the caregiver’s behaviour. In ignoring the caregiver’s needs the MHN feels he/she is acting in line with organizational policy. Explicit interventions in relation to the caregivers are not carried out.

The preventer considers the caregiver to be someone who plays an important role in the client’s care. Therefore, the focus is on preventing overburdening that might lead to a breakdown of the caregiving relationship. The burden is observed or presumed rather than openly discussed with the caregiver. In order to be effective, the preventer invests in building a relationship with the caregiver. However when this relationship conflicts with the client’s care needs, it is the client’s needs that take precedence. In addressing the caregiver’s needs, preventers mostly use practical solutions, which they feel are not always effective. Addressing emotional and relational problems is felt to be too complicated.

Concerners focus on wellbeing and not only on the consequences of the disease. They are concerned about client and caregiver as well as the client-caregiver relationship. They consider a supportive relationship to be their main tool, and listening and being present to be their most important intervention. In providing support, they do not try to solve problems themselves, but seek to empower the caregiver.

Our study enriches the dichotomous approach prevalent in studies concerning nurses’ attitudes to supporting family members [27]: the studies report that nurses either give attention to caregivers or not. The third prototype, the one in the middle, the preventer, is supportive to the family member in his or her caring role but does not focus on the wellbeing of the family member but on relieving the burden. In our view, the difference in approach between preventers and concerners cannot be overlooked.

Attention to caregivers’ needs confronts preventers and concerners with the boundaries of their competencies. Preventers avoid this confrontation by reducing their involvement, while concerners try to increase their competency or they make specific referrals.

Unlike the studies of [27–31] our study did not address the factors that determine whether MHNs are supportive or non-supportive. We found all three prototypes in the two organizations, across the different age groups and among nurses with greater or less experience or different functions. Neither of the organizations had an explicit family policy.

### Strengths and limitations

The use of constant comparative analyses and the intertwined use of coding and theoretical thinking, familiarity with the culture of the participating organizations, the purposeful sampling of the respondents, the strategies to foster free expressions of thoughts and data and researcher triangulation all contribute to the credibility of the research. Dependability is enhanced by a detailed description of the procedures followed during data collection. Confirmability is enhanced by triangulation and verification of results by an independent researcher with data not included in the analysis.

The personal and job characteristics of the participants were heterogeneous. However, they were recruited from only two large mental health care organizations. Moreover eleven of the 38 participating MHNs were working on the same division as the main researcher. This may have influenced the thoughts shared with the interviewer. Nonetheless, the interviews represented a broad spectrum of degree of concern regarding caregivers. Discussing their own practice and examples of their daily practice made it difficult to give socially desirable answers, and if this was the case, they were rather easy to detect.

As we described, the three types of MHN vary in their approach to caregiver support. The vision of the MHNs on caregiver support seems to direct the diversity in approach. This implies that a change in behaviour needs to be preceded by a change in vision. In keeping with the phases of change concept [32] we can say that the tolerator might be considered to be in a pre-contemplation phase. Interventions to support caregiving are not yet of interest to them. Hence, teaching skills in relation to these interventions will not be conductive to behavioural change. Preventers will need a change in vision from focusing on the consequences of disease to focusing on wellbeing. The concerners are in need of interventions that help to broaden the range of their skills to tackle more difficult situations and to evaluate the effect of their support [33]. Promoting family support cannot be achieved by a one-size-fits-all programme.

This study constitutes a first step in the field of MHN support to caregivers of older adults with severe mental illness. It is recommended that replication of the study should be conducted with other samples in psychiatry and other fields of nursing. As a follow up, further research is planned to address the following: first, identify factors that might explain how an MHN becomes one of the three prototypes; second, establish which intervention brings practice more in line with the desirable prototype; third, explore whether influencing the prototype improves the situation for both caregiver and client; fourth, examine the influence of the correlation between expectations and experienced needs on the one hand, and the caregiver’s behaviour on the other hand.
